# External validation of the international IgA nephropathy prediction tool in a Thai cohort

**DOI:** 10.3389/fmed.2026.1860001

**Published:** 2026-06-18

**Authors:** Atthaphong Phongphithakchai, Attamon Thongrueang, Thatsaphan Srithongkul, Sukit Raksasuk, Fumitaka Kawakami, Aman Tedasen, Moragot Chatatikun

**Affiliations:** 1 Nephrology Unit, Division of Internal Medicine, Faculty of Medicine, Prince of Songkla University, Hat Yai, Songkhla, Thailand; 2 Division of Nephrology, Department of Medicine, Faculty of Medicine, Siriraj Hospital, Mahidol University, Bangkok, Thailand; 3 Department of Regulatory Biochemistry, Graduate School of Medical Sciences, Kitasato University, Sagamihara, Japan; 4 Department of Medical Technology, School of Allied Health Sciences, Walailak University, Nakhon Si Thammarat, Thailand

**Keywords:** external validation, IgA nephropathy, prognostic model, risk stratification, Southeast Asia

## Abstract

**Objectives:**

To externally validate the performance of the International IgA Nephropathy Prediction Tool (IIgANPT) in a Thai cohort with biopsy-proven IgA nephropathy and to compare the predictive accuracy of model versions with and without the race variable.

**Methods:**

We conducted a retrospective cohort study to externally validate the IIgANPT in Thai adults with biopsy-proven IgAN at a tertiary-care center. Both model versions (with and without race) were evaluated. The primary outcome was a composite of a sustained 50% decline in estimated glomerular filtration rate (eGFR) or progression to end-stage renal disease (ESKD). Model performance was assessed using discrimination (C-statistic, AUC), calibration (slope and plots), and risk stratification with Kaplan–Meier analysis.

**Results:**

A total of 133 patients were included, with a median follow-up of 3.4 years. During follow-up, 45 patients (33.8%) reached the renal endpoint. The model demonstrated good discrimination, with C-statistics of 0.753 (95% CI 0.68–0.82) and 0.754 (95% CI 0.69–0.82) for models with and without race, respectively. Five-year AUC values were similar (0.790 vs. 0.787). Calibration was broadly preserved, although confidence intervals around calibration slopes were wide. Risk stratification showed clear separation of kidney survival across predefined risk groups (log-rank *p* < 0.0001).

**Conclusion:**

The IIgANPT demonstrated good discrimination and effective risk stratification in this Thai cohort of patients with biopsy-proven IgAN. Calibration point estimates were close to the ideal range, although precision was limited by wide confidence intervals. Both model versions performed similarly, suggesting that exclusion of the race variable may have limited impact on prognostic performance in this relatively homogeneous population.

## 1 Introduction

Immunoglobulin A nephropathy (IgAN) is the most common primary glomerulonephritis worldwide and remains an important cause of chronic kidney disease (CKD) and kidney failure (1, 2). Its clinical course is highly heterogeneous, ranging from isolated urinary abnormalities with preserved kidney function to progressive disease leading to end-stage kidney disease (ESKD). This marked variability in prognosis makes individualized risk stratification central to the management of patients with IgAN (3–7).

Over the past two decades, several clinicopathologic factors have been shown to be associated with adverse kidney outcomes in IgAN, including reduced estimated glomerular filtration rate (eGFR), higher proteinuria, elevated blood pressure, and chronic structural lesions on kidney biopsy. The Oxford Classification, particularly the MEST score, has become the most widely accepted histopathologic framework for prognostic assessment in IgAN and has been incorporated into routine pathology reporting and clinical research (8–11).

To facilitate individualized prognostication, Barbour and colleagues developed the International IgA Nephropathy Prediction Tool (IIgANPT), a multivariable model derived from a large, international, multiethnic cohort of patients with biopsy-proven IgAN (12). The model incorporates routinely available clinical variables at the time of biopsy, treatment-related variables, and Oxford MEST scores to estimate the risk of a 50% decline in eGFR or progression to kidney failure over 80 months. Two versions of the model are available: one including a race/ethnicity parameter and one excluding race12. The IIgANPT has been recognized as a useful adjunct for risk assessment in clinical practice and is referenced in the KDIGO 2021 Glomerular Diseases guideline (13).

However, prediction models must undergo external validation before they can be confidently applied outside the populations in which they were derived. Model performance may vary across settings because of differences in ethnicity, referral patterns, disease severity at biopsy, treatment practices, healthcare systems, and outcome frequency. Previous external validation studies across Chinese, European, and other Asian cohorts generally demonstrated preserved discrimination, although calibration performance varied and uncertainty remains regarding model transportability across different healthcare settings and ethnic populations (14–16). Therefore, additional validation in underrepresented populations is essential, in line with TRIPOD recommendations for prognostic model evaluation (17).

Importantly, clinical characteristics and treatment practices may differ substantially across populations. Compared with previously validated cohorts, Thai patients with IgAN may present with different disease severity at the time of kidney biopsy, referral patterns, and exposure to immunosuppressive therapy, all of which could influence model performance and transportability. Furthermore, the incremental value of the race parameter remains uncertain in relatively homogeneous Southeast Asian populations, where ethnic categorization may not fully align with those used in the original derivation study.

Data from Southeast Asia, especially Thailand, remain limited. Given potential differences in ethnicity, referral patterns, disease severity at biopsy, treatment practices, and healthcare systems, direct validation of the IIgANPT in Thai patients is needed before routine implementation. Accordingly, the present study aimed to externally validate the IIgANPT, both with and without the race parameter, in a Thai cohort of adults with biopsy-proven IgAN, and to evaluate its discrimination, calibration, and risk stratification performance.

## 2 Methods

### 2.1 Study design and setting

We conducted a retrospective cohort study to externally validate the International IgA Nephropathy Prediction Tool in Thai patients with biopsy-proven IgA nephropathy. The study was performed at Songklanagarind Hospital, a tertiary-care university hospital in southern Thailand. The protocol specified an external validation design focused on discrimination, calibration, and clinical utility of the published prognostic model.

### 2.2 Study population

Adult patients with biopsy-confirmed primary IgA nephropathy diagnosed between January 2015 and December 2024 were eligible for inclusion. Inclusion criteria comprised age 18 years or older at the time of kidney biopsy, biopsy-proven primary IgAN, availability of baseline clinical data required for model application, and documented Oxford MEST histologic scores. Patients were excluded if they had secondary IgA nephropathy, acute kidney injury unrelated to IgAN at the time of biopsy, baseline eGFR <15 mL/min/1.73 m^2^, prior chronic dialysis or kidney transplantation, incomplete key predictor data, or severe concomitant conditions expected to independently affect kidney prognosis.

### 2.3 Data collection and variables

Clinical and pathological data were retrospectively extracted from the hospital information system using a structured electronic data collection form. Baseline variables included age, sex, serum creatinine, eGFR, blood pressure, proteinuria, use of renin–angiotensin system blockade (RASB), use of immunosuppressive therapy, and Oxford MEST scores. eGFR was calculated using the CKD-EPI equation.

### 2.4 Outcome definition

The primary outcome for model validation was the composite renal endpoint defined as either a sustained 50% decline in eGFR from baseline or progression to ESKD. ESKD was defined as eGFR <15 mL/min/1.73 m^2^, initiation of chronic dialysis, or kidney transplantation. This outcome definition was prespecified in the protocol and is consistent with the original IIgANPT derivation study and published external validation work.

### 2.5 Model application and risk stratification

Predicted risks in this study were generated using the IIgANPT, applying both versions of the model: the model including race and the model excluding race. The implementation of the prediction model followed the framework described in the original study by Barbour et al. (12), in which individual risk estimates are calculated from baseline clinical and histopathological variables.

For subgroup analyses, patients were stratified into ordered risk categories based on the distribution of predicted risk scores. In line with the original study, patients with IgA nephropathy were classified into four predefined groups using percentile cut-offs: <16th percentile (low risk), 16th–50th percentile (intermediate risk), 50th–84th percentile (higher risk), and >84th percentile (highest risk). This classification allows comparison of outcomes across increasing levels of predicted risk.

### 2.6 Statistical analysis

Baseline characteristics were summarized using mean with standard deviation, median with interquartile range, or number with percentage, as appropriate. Between-group comparisons across risk strata used analysis of variance for normally distributed continuous variables, Kruskal–Wallis tests for non-normally distributed continuous variables, and chi-square or Fisher’s exact tests for categorical variables. Model performance was assessed in terms of discrimination, calibration, and risk-stratified survival analysis. Discrimination was evaluated using the concordance statistic (C-statistic) and the time-dependent area under the receiver operating characteristic curve (AUC) at 5 years. Because the primary outcome was time-to-event in nature, the 5-year AUC was estimated within a survival analysis framework to account for right-censored observations. Kaplan–Meier methods were used to evaluate outcome-free survival overall and across predefined risk groups. A two-sided *p* value < 0.05 was considered statistically significant. The analysis framework followed standard external validation principles and the TRIPOD recommendations.

## 3 Results

### 3.1 Study population and baseline characteristics

**Figure 1:**
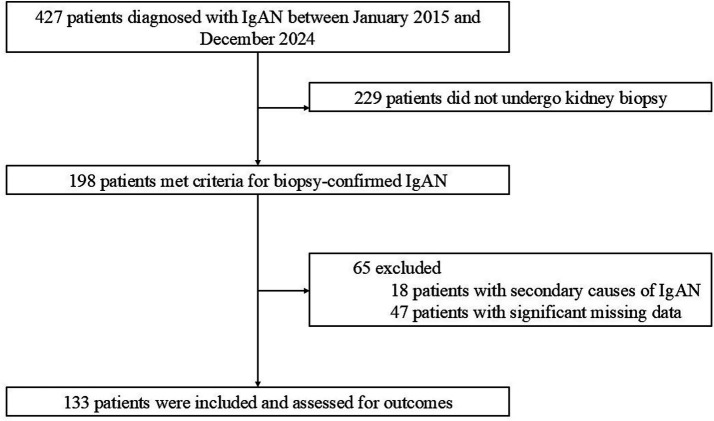
Study flow chart.

A total of 427 patients diagnosed with IgA nephropathy between January 2015 and December 2024 were initially screened. Of these, 229 patients who did not undergo kidney biopsy were excluded. Among the remaining 198 patients with biopsy-confirmed IgA nephropathy, 65 were further excluded, including 18 patients with secondary causes of IgAN and 47 patients with significant missing data. Ultimately, 133 patients with biopsy-proven primary IgA nephropathy were included in the final analysis (Figure 1).

A total of 133 patients with biopsy-proven IgA nephropathy were included in the analysis. The median follow-up duration was 3.4 years (IQR 0.5–6.8). Baseline characteristics of the study population are summarized in Table 1, alongside comparisons with the original derivation and original validation cohorts of the IIgANPT. Overall, the cohort demonstrated a broad spectrum of disease severity at the time of kidney biopsy. Patients in higher-risk groups tended to be older and had progressively higher blood pressure, lower baseline eGFR, and greater proteinuria, indicating worsening kidney disease severity with increasing predicted risk (all *p* < 0.001). Compared with the original derivation and validation cohorts, our cohort also differed in several clinical characteristics and treatment patterns, including a higher proportion of immunosuppressive therapy exposure and more advanced histopathologic lesions.

**Table 1:** Baseline characteristics across the external validation, original derivation, and original validation cohorts.

Variable	Original derivation cohort (*n* = 2,781)	Original validation cohort (*n* = 1,146)	External validation cohort in this study (*n* = 133)
Follow-up time, years	4.8 (3.0–7.6)	5.8 (3.4–8.5)	3.4 (0.5–6.8)
Age, years	35.6 (28.2–45.4)	34.8 (26.9–45.0)	41.8 (13.3)
Male	1,608 (57.8)	565 (49.3)	59 (44.4)
Proteinuria at biopsy, g/day			
<0.49	383 (13.9)	221 (19.4)	51 (38.3)
0.5–1.0	772 (28.1)	209 (18.3)	47 (35.3)
1.0–2.0	817 (29.7)	352 (30.8)	19 (14.3)
>2.0	775 (28.2)	360 (31.5)	16 (12.0)
eGFR, mL/min/1.73 m^2^			
<30	142 (5.1)	37 (3.2)	51 (38.3)
30–59	657 (23.6)	191 (16.7)	47 (35.3)
60–90	800 (28.8)	350 (30.5)	19 (14.3)
>90	1,182 (42.5)	568 (49.6)	16 (12)
Oxford classification			
M1	1,054 (38.0)	481 (42.0)	65 (48.9)
E1	478 (17.3)	476 (41.5)	0 (0)
S1	2,137 (77.0)	912 (79.6)	117 (88.0)
T1	686 (24.7)	207 (18.1)	57 (42.9)
T2	128 (4.6)	122 (10.6)	31 (23.3)
C1	953 (34.3)	642 (56.1)	22 (16.5)
RAAS-blockage	2,400 (86.7)	708 (66.4)	73 (54.9)
Immunosuppressive drug	1,209 (43.5)	359 (31.3)	103 (77.4)
Primary outcome			
50% decline in eGFR	420 (15.1)	210 (18.3)	32 (24.1)
ESKD	372 (13.4)	155 (13.5)	45 (33.8)

Continuous data are demonstrated as median (IQR); categorical data are demonstrated as count (percentage). eGFR, estimated glomerular filtration rate; ESKD, end-stage kidney disease; RAAS, renin–angiotensin–aldosterone system; M, mesangial hypercellularity; E, endocapillary hypercellularity; S, segmental glomerulosclerosis; T, tubular atrophy/interstitial fibrosis; C, cellular/fibrocellular crescents.

Histopathological findings based on the Oxford classification also demonstrated significant differences across risk groups. The proportion of patients with mesangial hypercellularity (M1) increased steadily from the low-risk to the highest-risk group. A similar trend was observed for segmental sclerosis (S1), which was highly prevalent in higher-risk categories and reached nearly universal presence in the highest-risk group. Tubulointerstitial damage, represented by the T score, showed the most pronounced gradient, with minimal involvement (T0) in the low-risk group and a marked increase in T1 and T2 lesions in higher-risk groups (*p* < 0.001). Endocapillary hypercellularity (E1) did not show a consistent statistically significant difference across groups. The presence of crescents was relatively comparable across risk categories without significant variation.

Further detailed comparisons of clinical and histopathologic characteristics across risk groups are presented in Table 2. Both models demonstrated highly consistent patterns. Increasing risk categories were associated with significantly higher blood pressure, lower eGFR, and greater proteinuria (all *p* < 0.001). The distribution of Oxford MEST scores was also similar between the two models, confirming that disease severity increased in parallel with predicted risk regardless of inclusion of the race variable. Notably, the gradients of eGFR decline and proteinuria escalation across risk groups were nearly identical in both models, suggesting that exclusion of the race parameter did not materially affect risk stratification in this cohort. During the follow-up period, 45 patients (33.8%) progressed to ESKD, while 88 patients (66.2%) did not reach the renal endpoint.

**Table 2:** Clinical and histopathological characteristics stratified by four risk subgroups.

Character	Low risk (*n* = 22)	Intermediate risk (*n* = 45)	High risk (*n* = 44)	Highest risk (*n* = 22)	*p* value
Age, years	31.2 ± 9.1	43.8 ± 14.5	45.4 ± 11.8	41 ± 12.3	<0.001
SBP, mmHg	120 (113.2–124.5)	130 (117–140)	134.5 (120–144.2)	126 (119–141.5)	0.009
DBP, mmHg	80 (72.5–82)	80 (72–90)	80.5 (71.8–90)	72.5 (67.8–87)	0.515
eGFR, mL/min/1.73 m^2^					<0.001
<30	0 (0)	0 (0)	29 (65.9)	22 (100)	
30–59	0 (0)	32 (71.1)	15 (34.1)	0 (0)	
60–90	6 (27.3)	13 (28.9)	0 (0)	0 (0)	
>90	16 (72.7)	0 (0)	0 (0)	0 (0)	
Proteinuria, g/day	0.9 (0.4–2.3)	1.2 (0.6–2.4)	2.2 (1.4–2.8)	2.5 (1.5–4.3)	
Oxford classification					
M score					0.004
M0	19 (86.4)	20 (44.4)	18 (40.9)	11 (50)	
M1	3 (13.6)	25 (55.6)	26 (59.1)	11 (50)	
E score					0.002
E0	22 (100)	45 (100)	44 (100)	22 (100)	
S score					0.447
S0	5 (22.7)	5 (11.1)	4 (9.1)	2 (9.1)	
S1	17 (77.3)	40 (88.9)	40 (90.9)	20 (90.9)	
T score					<0.001
T0	18 (81.8)	15 (33.3)	10 (22.7)	2 (9.1)	
T1	4 (18.2)	25 (55.6)	18 (40.9)	10 (45.5)	
T2	0 (0)	5 (11.1)	16 (36.4)	10 (45.5)	
C score					0.55
C0	19 (86.4)	39 (86.7)	37 (84.1)	16 (72.7)	
C1	3 (13.6)	6 (13.3)	7 (15.9)	6 (27.3)	

Continuous data are demonstrated as mean ± SD and median (IQR); categorical data are demonstrated as count (percentage). eGFR, estimated glomerular filtration rate; DBP, diastolic blood pressure; SBP, systolic blood pressure.

### 3.2 Model performance

**Figure 2:**
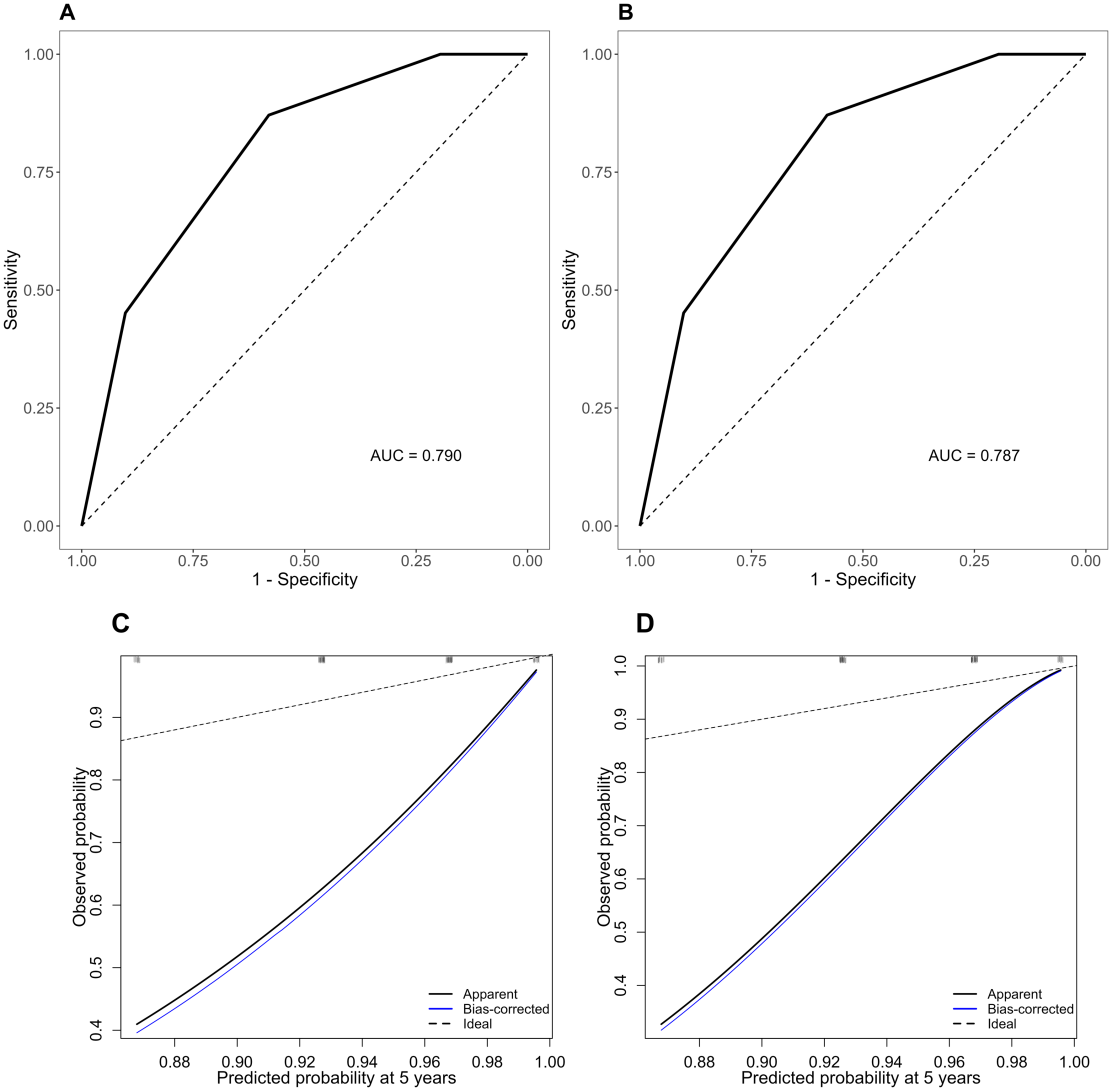
Performance of the International IgA Nephropathy Prediction Tool (IIgANPT) for predicting renal outcomes: receiver operating characteristic (ROC) curves for the **(A)** model with race and **(B)** model without race, and calibration plots of predicted versus observed risk for the **(C)** model with race and **(D)** model without race.

The predictive performance of the IIgANPT was evaluated in terms of discrimination and calibration. The concordance statistic (C-statistic) was 0.753 (95% CI 0.68–0.82) for the model including race and 0.754 (95% CI 0.69–0.82) for the model excluding race. The area under the receiver operating characteristic curve (AUC) at 5 years was 0.790 for the model with race and 0.787 for the model without race. The ROC curves for both models demonstrated similar shapes across the full range of sensitivity and specificity, with no apparent separation between the two models (Figure 2).

**Figure 3:**
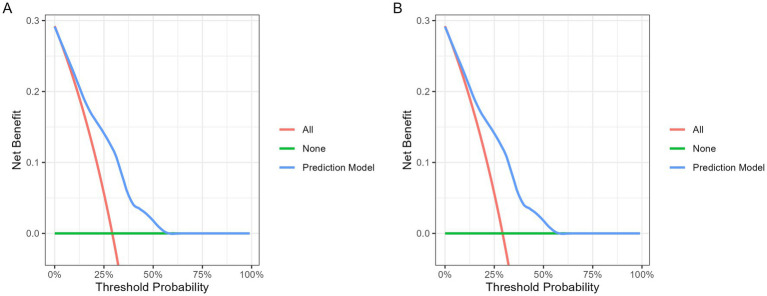
Decision curve analysis (DCA) of the International IgA Nephropathy Prediction Tool (IIgANPT) for prediction of renal outcomes: **(A)** model with race and **(B)** model without race.

Calibration was assessed using calibration slope and graphical evaluation. The calibration slope was 1.05 (95% CI 0.520–1.671) for the model including race and 1.01 (95% CI 0.513–1.669) for the model excluding race. Although point estimates were close to the ideal value of 1, confidence intervals were relatively wide, suggesting limited precision of calibration estimates. In the calibration plots, the apparent and bias-corrected curves were generally aligned with the ideal reference line across most of the prediction range, with minor deviations observed at lower predicted probabilities. Similar patterns were observed in both models (Figure 2). Decision curve analysis demonstrated positive net benefit for both IIgANPT models across clinically relevant threshold probabilities when compared with treat-all and treat-none strategies. The net benefit curves of the models with and without race were highly similar, with no meaningful separation observed between model versions, suggesting comparable potential clinical utility (Figure 3).

**Figure 4:**
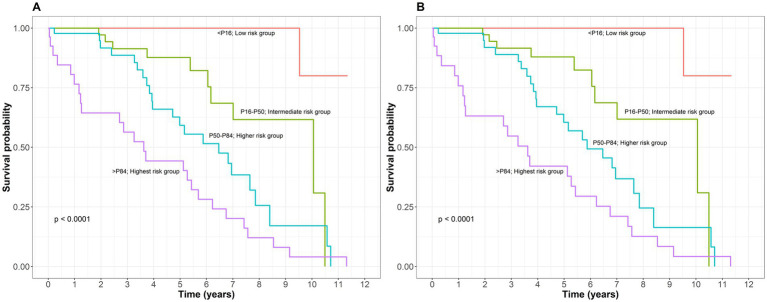
Kaplan–Meier curves of kidney survival according to risk stratification by the International IgA Nephropathy Prediction Tool (IIgANPT): **(A)** model with race variable and **(B)** model without race variable.

Risk stratification based on predicted probabilities divided patients into four predefined groups: low risk (<16th percentile), intermediate risk (16th–50th percentile), higher risk (50th–84th percentile), and highest risk (>84th percentile). Kaplan–Meier survival analysis demonstrated a stepwise separation of survival curves across these groups, with progressively lower kidney survival observed in higher risk categories. The separation between curves was evident early during follow-up and persisted over time. The differences between groups were statistically significant (log-rank *p* < 0.0001) for both the model including race and the model excluding race. The relative ordering of risk groups was preserved across both models, with similar curve patterns and spacing (Figure 4).

Given the high proportion of immunosuppressive therapy exposure in our cohort, an exploratory subgroup analysis was performed to evaluate model performance among treated patients. In this subgroup, both model versions demonstrated similar performance. The concordance statistics were 0.753 (95% CI 0.68–0.82) for the model with race and 0.754 (95% CI 0.69–0.82) for the model without race, while the 5-year AUC values were 0.741 and 0.754, respectively. Calibration slope point estimates were below 1, with wide confidence intervals [0.80 (95% CI 0.27–1.48) for the model with race and 0.83 (95% CI 0.30–1.60) for the model without race], suggesting limited precision of calibration estimates. Detailed subgroup analyses are provided in the Supplementary Figures 1 and 2.

## 4 Discussion

In this external validation study of the IIgANPT in a Thai cohort, both models (with and without race) demonstrated similar performance across discrimination, calibration, and risk stratification analyses. The C-statistics were comparable between models, and the 5-year AUC values were nearly identical. Calibration slope point estimates were close to the ideal value of 1, although confidence intervals were relatively wide, reflecting limited precision of calibration estimates. Calibration plots showed broadly similar patterns between predicted and observed risks across most of the prediction range. In addition, risk stratification based on predicted probabilities showed clear and progressive separation of renal outcomes across predefined risk groups, with consistent patterns observed in both models.

Importantly, our findings extend the evidence base for the IIgANPT to a Southeast Asian population, a region that has remained underrepresented in prior validation studies. Although overall predictive performance was broadly consistent with previous reports from Chinese and European cohorts, our study population differed in several clinically relevant aspects that may influence model transportability. Specifically, our cohort was derived from a tertiary referral center, demonstrated a relatively high burden of chronic histopathologic lesions, and had a notably high proportion of immunosuppressive therapy exposure. These differences highlight the importance of validating prognostic models within distinct healthcare systems and treatment contexts before widespread implementation in routine clinical practice.

The performance observed in this study is broadly consistent with prior external validation studies of the IIgANPT conducted across different populations. In a Chinese cohort by Hu et al., the model demonstrated strong discriminative ability, with AUC values of approximately 0.88 at 5 years, along with acceptable calibration and positive net clinical benefit based on decision curve analysis (15). Similarly, Du et al. externally validated the updated IIgANPT in a Chinese cohort and demonstrated preserved discrimination; however, the model showed limited ability to distinguish between low- and intermediate-risk groups, highlighting constraints in risk stratification despite improved calibration (16).

The original derivation and validation study by Barbour et al. showed that the IIgANPT maintained predictive performance across multiethnic populations, including Asian and European cohorts, supporting its generalizability (12). Subsequent studies have confirmed that the model performs consistently across different clinical settings, with preserved discrimination; however, variation in calibration has been reported, likely reflecting differences in baseline risk, treatment patterns, and case-mix across populations (18).

Several studies have specifically evaluated the role of the race variable in the IIgANPT. In Asian populations, prior validations suggest that exclusion of the race parameter does not substantially alter discrimination, while its impact on calibration appears to be modest and context-dependent (14, 15). Zhang et al. demonstrated that the model without race maintained comparable discrimination in an Asian cohort, although minor differences in calibration were observed (19). A recent external validation in a French cohort demonstrated comparable discrimination and generally acceptable calibration for both models, although some divergence in long-term predictions was noted when ethnicity was omitted (20). The role of race in the original IIgANPT may reflect a composite surrogate for differences in disease phenotype, healthcare systems, treatment access, and baseline risk rather than biological ethnicity alone. Although Pacific Asian ancestry was associated with disease progression in the original derivation cohort, important heterogeneity exists across Asian subpopulations. Compared with East Asian cohorts, Southeast Asian populations may differ in referral pathways, timing of kidney biopsy, immunosuppressive prescribing practices, and healthcare accessibility, all of which could influence model transportability. In the present study, the nearly identical performance of models with and without race suggests that the race parameter provides limited incremental prognostic information in this relatively ethnically homogeneous Thai cohort. Nevertheless, further multicenter and multinational validation studies in Southeast Asia are warranted to confirm these findings and explore whether regional recalibration or model updating may improve predictive performance.

Nevertheless, interpretation of calibration performance should be cautious. Although point estimates of the calibration slope were close to the ideal value of 1, confidence intervals were relatively wide, likely reflecting the modest sample size and limited number of outcome events. Therefore, while the findings suggest broadly preserved calibration, statistical uncertainty remains and larger multicenter studies are needed to confirm calibration precision^16^.

The ability of the IIgANPT to stratify patients into distinct risk categories has direct clinical relevance. Risk-based classification can assist clinicians in identifying patients at higher risk of disease progression who may benefit from closer monitoring, more aggressive supportive care, or consideration of immunosuppressive therapy, while avoiding overtreatment in low-risk individuals. In addition, the comparable performance of models with and without the race variable suggests that the simplified model may be sufficient for clinical use in settings where race classification is not applicable or may introduce ambiguity. This is particularly relevant in Southeast Asian populations, where ethnic categorization may not align with the original model definitions. Furthermore, external validation in a real-world Thai cohort supports the applicability of the IIgANPT in routine clinical practice, including integration into clinical decision-making and potentially into electronic health record–based risk calculators.

This study has several strengths. First, it represents one of the first external validation studies of the IIgANPT in a Southeast Asian population, addressing an important gap in the literature where data from this region remain limited. Second, the study was conducted in a real-world clinical setting using routinely collected data, enhancing the practical relevance and applicability of the findings to everyday clinical practice. Third, comprehensive clinical and histopathological data were available, allowing full implementation of the original model, including all required predictors such as proteinuria, eGFR, blood pressure, treatment variables, and Oxford MEST scores. Fourth, multiple domains of model performance were evaluated, including discrimination, calibration, and risk stratification, in accordance with recommendations from the TRIPOD statement and contemporary guidelines for prediction model validation. The inclusion of both models (with and without the race variable) allowed for a direct comparison of model performance and provided additional insight into the relevance of the race parameter in a relatively homogeneous population. In addition, internal validation using bootstrap methods was performed to assess the stability of calibration estimates, reducing the risk of overfitting.

However, several limitations should be considered. First, the retrospective design introduces potential selection bias and limits the ability to control for unmeasured confounders. Second, this was a single-center study conducted at a tertiary referral hospital, which may limit generalizability to other healthcare settings, particularly primary or secondary care environments with different patient characteristics. Third, the sample size and number of outcome events were relatively limited, which may affect the precision and stability of model performance estimates, particularly calibration metrics. The wide confidence intervals observed around calibration slope estimates highlight the statistical uncertainty inherent in a modestly sized external validation cohort and warrant cautious interpretation of calibration performance. Fourth, the median follow-up duration was relatively limited for a disease characterized by slow progression, with a median follow-up of 3.4 years. Although a subset of patients had follow-up extending beyond 5 years, allowing estimation of model performance at the 5-year time point, the observed follow-up period may not fully capture the long-term disease trajectory of IgA nephropathy, which often evolves over decades. Fifth, although the model includes key clinical and histopathologic predictors, other potentially relevant variables such as time-updated proteinuria, longitudinal eGFR slope, or novel biomarkers were not incorporated in this validation. Finally, external validation was performed in a relatively homogeneous ethnic population, which, while appropriate for evaluating the role of the race variable, may limit extrapolation to more diverse populations. Further multicenter and multinational validation studies are needed to confirm these findings and to explore potential model updating or recalibration strategies in different clinical settings.

In conclusion, in this external validation study, the International IgA Nephropathy Prediction Tool demonstrated good discrimination and effective risk stratification in a Thai cohort of patients with biopsy-proven IgAN. Calibration point estimates were close to the ideal range, although precision was limited by wide confidence intervals. Both model versions, with and without the race variable, showed comparable performance, suggesting that exclusion of race may have limited impact on prognostic performance in this relatively homogeneous population. These findings support the applicability of the IIgANPT for risk assessment in Southeast Asian clinical settings, while further multicenter validation is warranted.

## Data Availability

The raw data supporting the conclusions of this article will be made available by the authors, without undue reservation.
