# Supplementary material for: External validation of the international IgA nephropathy prediction tool in a Thai cohort
Source: Front Med (Lausanne). 2026 Jun 18;13:1860001. doi: 10.3389/fmed.2026.1860001 (PMC13322946; doi:10.3389/fmed.2026.1860001)

Supplementary Material

**Supplementary Figure 1.** Performance of the International IgA Nephropathy Prediction Tool (IIgANPT) for predicting renal outcomes among patients receiving immunosuppressive therapy: receiver operating characteristic (ROC) curves for the (A) model with race and (B) model without race, and calibration plots of predicted versus observed risk for the (C) model with race and (D) model without race.


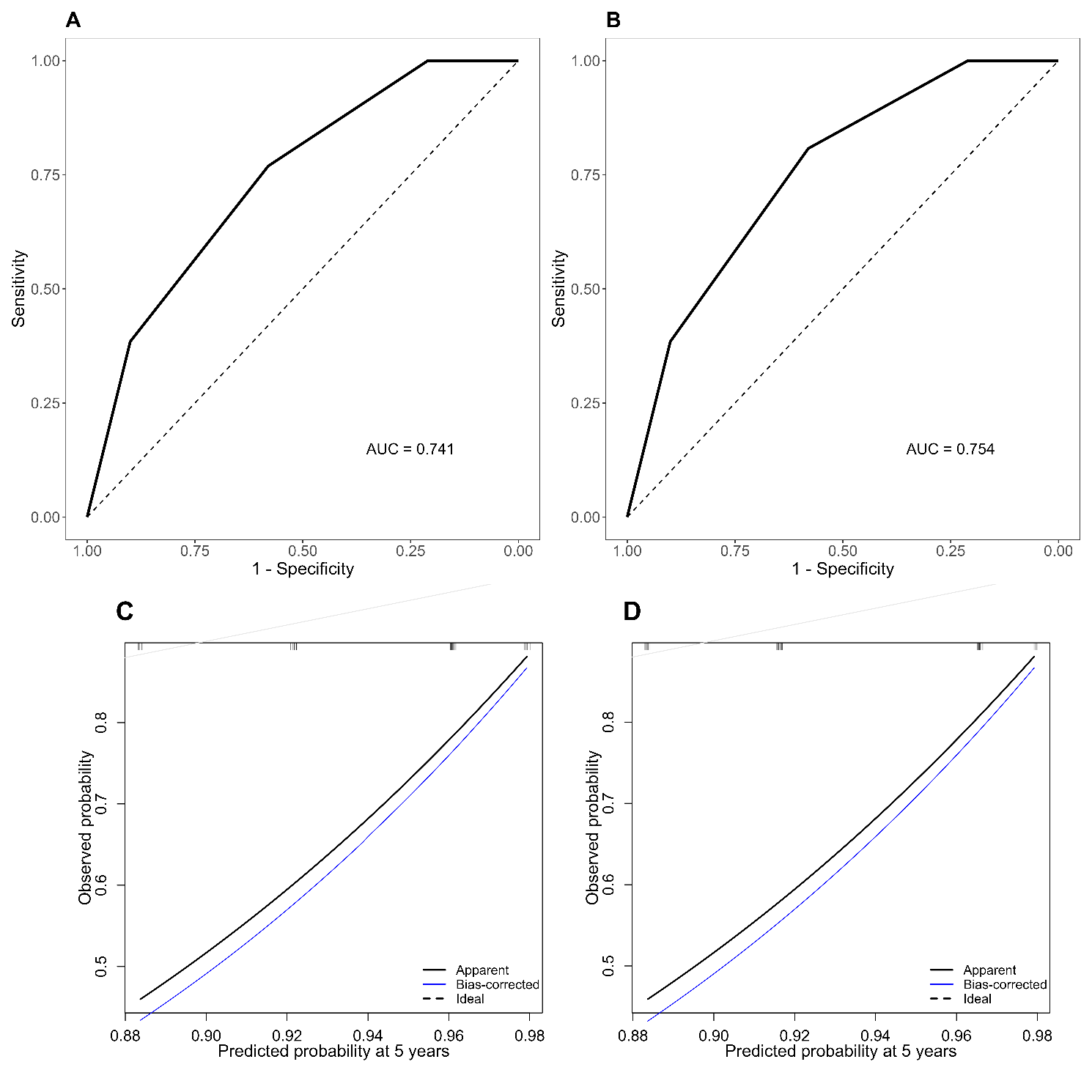


**Supplementary Figure 2**. Decision curve analysis (DCA) of the International IgA Nephropathy Prediction Tool (IIgANPT) for prediction of renal outcomes among patients receiving immunosuppressive therapy: (A) model with race and (B) model without race.


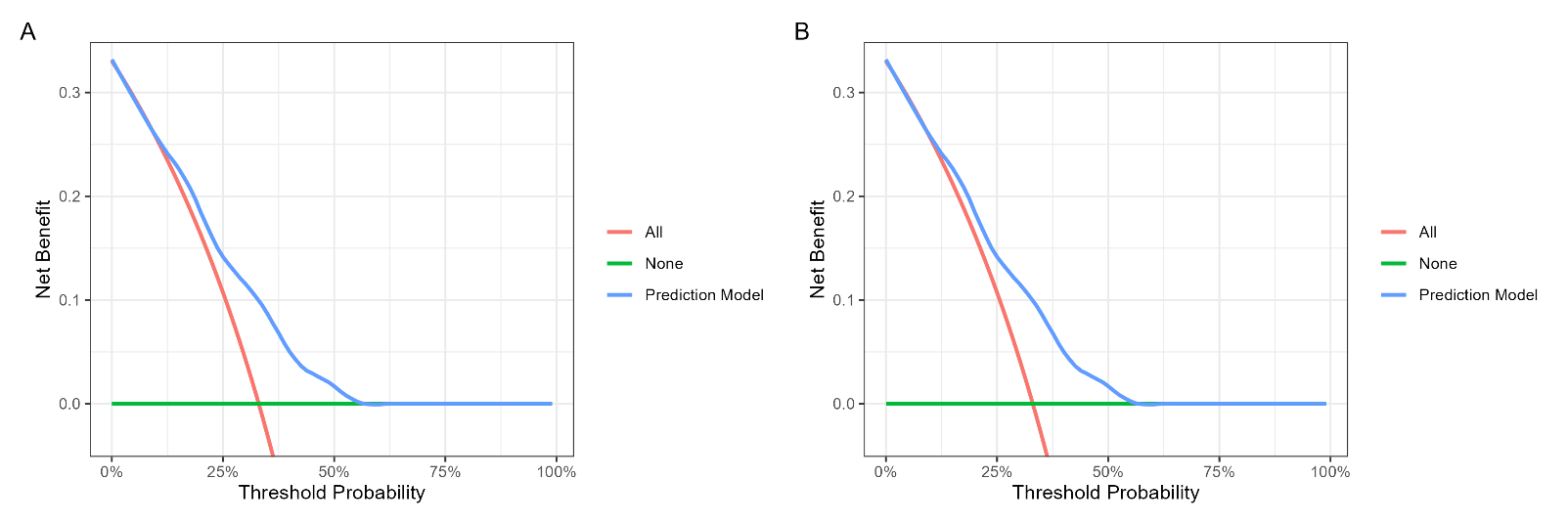

Supplement: Supplementary file 1 [file Table_1.DOCX]
